# Types and Characteristics of Hair Across the Globe: Results of a Multinational Study on 19,461 Individuals

**DOI:** 10.2147/CCID.S377009

**Published:** 2025-02-18

**Authors:** Frederic Flament, Aurelie Maudet, Philippe Barbarat, Meriem Jabnoun, Muriel Bayer-Vanmoen, Audrey Imbert legrain, Stephanie Leclerc-Mercier, Charles Taieb, Charbel Skayem, Laurence Lebarbanchon

**Affiliations:** 1 L’Oréal Research and Innovation, Clichy, France; 2 L’Oréal Research and Innovation, Aulnay-sous-Bois, France; 3 L’Oréal, Levallois-Perret, France; 4 Laboratoires Vichy, Levallois Perret, France; 5 Patients Priority Dpt, EMMA, Paris, France; 6 Sorbonne University, Faculty of Medicine, Paris, France; 7 Hôpitaux de Paris (AP-HP), Paris Saclay University, Ambroise Paré Hospital, Boulogne Billancourt, France

**Keywords:** hair, impact on daily life, type, countries, region, quality of life

## Abstract

**Introduction:**

Hair plays a critical role in enhancing physical appearance, self-esteem, and identity. However, the impact of hair characteristics on daily life has received limited attention. This study aimed to assess the types and characteristics of hair, their variations according to age, gender, ethnicity, and country, and their effects on individuals’ daily lives.

**Methods:**

A study was conducted between February 2022 and June 2023 across nine countries: USA, France, Brazil, India, China, Mexico, Japan, South Africa, and Indonesia. A total of 19,461 participants aged 18 and older were selected using stratified, proportional sampling. Participants completed a structured digital questionnaire evaluating hair thickness, type, damage, and curl degree, alongside questions on stress, sleep, and well-being. Data were analyzed using descriptive statistics and statistical tests (*T*-test, Pearson’s test).

**Results:**

Thin hair was reported by 23.7% of males and 29.2% of females. Dry hair was more common in females (38.9%) than males (32.1%). Damaged hair was reported by 47% of females and 30% of males. Ethnic differences showed that thick hair was most common in African (40.7%) and least in Asian (26.3%) populations. The impact of hair type on daily life varied significantly across countries. Individuals with damaged or dry hair experienced higher stress, lack of sleep, and poor well-being, especially in Japan, India, and France. Hair type (straight, wavy, curly, kinky) also affected personal and professional stress levels differently in each country.

**Conclusion:**

Our research shows that hair characteristics significantly influence daily life, self-image, and well-being, with notable variations by gender, ethnicity, and country. These findings highlight the need for interventions to address the psychological and social impacts of hair issues, contributing to better healthcare, body image, and product development.

## Introduction

Hair is considered to be one of the most important components of the beauty care processes and tends to attract the use of a variety of hair treating cosmetics.^1^ In fact, hair holds significant importance in the realm of beauty. It enhances physical appearance, expresses individuality and style, boosts confidence and self-esteem, and is a symbol of youthfulness and vitality.

In the field of dermatology, assessment of the impact on daily life is becoming an increasing interest in studies^2–6^ and remains important to estimate the burden of patients with skin conditions.^7^,^8^ Since hair beauty complements skin beauty by creating a cohesive and harmonious overall look that enhances natural beauty, it is equally important to evaluate the effect of hair conditions on daily life of individuals.

However, the impact of different hair types and characteristics on daily life of individuals has so far received minimal attention. Therefore, our objective was to perform the first international study that assesses types and characteristics of hair according to age, gender, ethnicity, and country, as well as their impact on daily life.

## Materials and Methods

### Population Selection

The survey was conducted by a polling company between February 2022 and June 2023 in USA [n=2512], France [n=1617], Brazil [n=2073], India [n=2960], China [n=2603], Mexico [n=2292], Japan [n=451], South Africa [n=2698] and Indonesia [n=2255].

The studied population was constituted of individuals aged 18 years and above, with around 3/4 females and 1/4 males, as this study was part of a larger international project on hair that focused on females, explaining the difference in proportions regarding gender. However, each of the male and female population of the study was a representative sample of the male and female population in each of the included countries. In fact, the selection used a stratified, proportional sampling with a replacement design.

In each of the countries in which it was conducted, proportional quota sampling was used based on the distribution of the population according to age, phototype, environment (large cities, towns, and rural areas), and income, in order to guarantee representativeness of the sample in each of the male and female group.

The eligibility of a respondent was based on demographic data, which were used to create a quota‐based sample. When a quota was filled, subsequent people in this category were no longer eligible.

Selected participants from all the respective countries were contacted by personal Email inviting them to take part in a survey without any specification of the subject of the survey. This prevented a self‐selection bias since participants with hair problems or concerns could have been more prone to participate in the study if its purpose had been disclosed. If contact was not successful, another potential participant with the same sociodemographic characteristics was randomly selected. To ensure the robustness of the data collected, individuals who did not complete the whole survey were excluded.

### Questionnaire and Outcomes

Participants were asked to complete a structured digital questionnaire that evaluated patients’ 1) hair thickness: thick, thin, or no specificities, 2) hair type: dry, oily, or no specificities, 3) hair damage state: damaged or not, as well presence of some characteristics: frizzy due to humidity, difficult to smoothen, badly defined curls, too much volume, low density, split ends, dull without shine, hair loss, baldness 4) curl degree: straight, wavy, curly, and kinky. In order to specify the curl degree, participants were provided with a visual scale that represents the different curl degrees. We compared these types and characteristics according to age, gender, country and ethnicity (European, African, Hispanic, Asian, and other) (in countries where legislation permits to ask about ethnicity). We note that individuals in a same country can have different ethnicities and the same individual can have multiple ethnicities. We also asked participants whether or not they feel that they are stressed in their personal or professional life, tired, lack sleep, confidence, or cheerfulness, feel bad about themselves. We compared the impact on daily life according to thickness (thick vs thin), hair type (dry vs oily), hair damage state (damaged vs not damaged), damaged hair according to gender (damaged hair in males vs females), and curl degree (straight or wavy, curly, and kinky). Moreover, for damaged, oily, dry, and different curl degrees, we compared the impact on daily life among the different studied countries.

The questionnaire for data collection was designed in English by the scientific committee of the project which includes expert dermatologists and public health specialists. Once created, the questionnaire was administered to a small sample to ensure that they were properly understood. It was then translated into each language by native speakers living in the country concerned. To ensure linguistic similarity and cultural coherence between different language versions, the translations produced by native speakers were then “back‐translated” in English.

### Statistical Analysis

Qualitative and ordinal variables were described by their number and frequency.

Results were tabulated in Excel and subsequently analysed. The student’s two-tailed *T*-test was used to determine statistical significance between groups. Pearson’s test was used to determine correlation. Results were considered statistically significant at a p-value <0.05.

The statistical analyses were carried out using the HARMONIE 1.7 software, registered with the INPI under the name DSE‐HARMONIE since 25 April 2013 with the registration number 4000937.

### Ethics

The study has the following IRB number: 2023-A02701-44.

All participants provided informed consent, in accordance with the Declaration of Helsinki.

## Results

In total, there were 19,461 participants in the study, of which 14317 (73.6%) females and 5138 (26.4%) males. The mean age was 39.7±13 y.o [median 40].

Thin hair was reported in 23.7% of males vs 29.2% of females (p <10^−3^). Thick hair was reported in 31.4% of males and females (p=0.9). Dry hair was reported in 32.1% of males vs 38 0.9% of females (p <10^−3^). Oily hair was reported in 12.9% of males vs 11.1% of females (p <10^−3^). Damaged hair was reported in 47% of females vs 30% of males (p <10^−3^). The comparison of types and characteristics of hair according to age and gender are represented in Table 1. Hair types and characteristics based on ethnicity are represented in Table 2. Thick hair was reported in 40.7% of African ethnicity and 26.3% of Asian ethnicity. Thin hair was reported in 34.7% of European ethnicity and 23.3% of Asian ethnicity. Dry hair was found in 46.7% of African, 33.2% of European, 35.8% of Hispanic, and 35.1% of Asian ethnicity. Damaged hair was reported in 47.6% of Asian ethnicity and 34.7% of European ethnicity. The impact on daily life of individuals according to hair thickness, oily/dry types, hair damage state, and curl degree is represented in Table 3.

**Table 1:** Characteristics of Hair Based According to Age and Gender

	n	Female n=14317		Male n=5138		p value	<40 y n=9622		≥40 y n=9839		p value
		n	%	n	%		n	%	n	%	
Thick	6104	4489	31.4%	1615	31.4%	0.931	3440	35.8%	2664	27.1%	0.000
Thin	5401	4184	29.2%	1217	23.7%	0.000	2268	23.6%	3133	31.8%	0.000
Dry	7212	5562	38.8%	1650	32.1%	0.000	3345	34.8%	3867	39.3%	0.000
Oily	2258	1595	11.1%	663	12.9%	0.001	1245	12.9%	1013	10.3%	0.000
Damaged	8266	6723	47.0%	1543	30.0%	0.000	4234	44.0%	4032	41.0%	0.000
Frizzy due to humidity	2703	2064	14.4%	639	12.4%	0.000	1285	13.4%	1418	14.4%	0.000
Difficult to smoothen	1801	1363	9.5%	438	8.5%	0.037	918	9.5%	883	9.0%	0.000
Badly defined curls	1566	1168	8.2%	398	7.7%	0.368	770	8.0%	796	8.1%	0.000
Too much volume	2055	1539	10.7%	516	10.0%	0.165	1087	11.3%	968	9.8%	0.000
Low density	2066	1544	10.8%	522	10.2%	0.222	966	10.0%	1100	11.2%	0.000
Split ends	3272	2533	17.7%	739	14.4%	0.000	1631	17.0%	1641	16.7%	0.000
Dull without shine	2028	1538	10.7%	490	9.5%	0.016	956	9.9%	1072	10.9%	0.000
Hair loss	10470	7964	55.6%	2506	48.8%	0.000	5122	53.2%	5348	54.4%	0.000
Bald	364	198	1.4%	166	3.2%	0.000	172	1.8%	192	2.0%	0.000
CURL											
Straight	6355	4285	29.9%	2068	40.2%	0.000	3027	31.5%	3328	33.8%	0.000
Wavy	8092	6354	44.4%	1737	33.8%	0.000	3992	41.5%	4100	41.7%	0.796
Curly	2487	2042	14.3%	443	8.6%	0.000	1269	13.2%	1219	12.4%	0.000
Kinky	2526	1636	11.3%	890	17.3%	0.000	1334	13.9%	1192	12.1%	0.000

**Table 2:** Characteristics of Hair Based on Ethnicity

	n	European n=3480		African n=3468		Hispanic n=3009		Asia n=8457		Others n=1949	
	Population	n	%	n	%	n	%	n	%	n	%
Thick	6104	1135	32.6%	1411	40.7%	1010	33.6%	2219	26.2%	630	32.3%
Thin	5401	1207	34.7%	867	25.0%	946	31.4%	1968	23.3%	721	37.0%
Dry	7212	1153	33.1%	1617	46.6%	1077	35.8%	2964	35.0%	723	37.1%
Oily	2258	333	9.6%	324	9.3%	320	10.6%	1122	13.3%	227	11.6%
Damaged	8266	1205	34.6%	1181	34.1%	1328	44.1%	4028	47.6%	826	42.4%
Frizzy due to humidity	2703	847	24.3%	363	10.5%	377	12.5%	934	11.0%	428	22.0%
Difficult to smoothen	1801	324	9.3%	403	11.6%	248	8.2%	744	8.8%	174	8.9%
Badly defined curls	1566	390	11.2%	230	6.6%	307	10.2%	526	6.2%	246	12.6%
Too much volume	2055	400	11.5%	561	16.2%	503	16.7%	388	4.6%	320	16.4%
Low density	2066	389	11.2%	222	6.4%	185	6.1%	1153	13.6%	208	10.7%
Split ends	3272	730	21.0%	510	14.7%	560	18.6%	1237	14.6%	449	23.0%
Dull without shine	2028	455	13.1%	226	6.5%	383	12.7%	801	9.5%	264	13.5%
Hair loss	10470	1838	52.8%	1387	40.0%	1662	55.2%	5031	59.5%	1014	52.0%
Bald	364	42	1.2%	112	3.2%	58	1.9%	137	1.6%	30	1.5%
CURL											
Straight	6355	1094	31.4%	1143	33.0%	975	32.4%	2835	33.5%	605	31.0%
Wavy	8092	1493	42.9%	1441	41.6%	1155	38.4%	3588	42.4%	769	39.5%
Curly	2488	423	12.2%	417	12.0%	419	13.9%	1066	12.6%	293	15.0%
Kinky	2526	470	10.5%	466	10.4%	460	11.5%	968	11.4%	282	14.5%

**Table 3:** Impact on Daily Life of Individuals According to Hair Thickness, Types, Hair Damage State, and Curl Degree

			Stress Level in Personal Life		Tiredness		Lack Sleep		Stress Level in Professional Life		Lack Energy		Lack Confidence		Feel Bad About Myself		Lack Cheerfulness	
			N	%	N	%	N	%	N	%	N	%	N	%	N	%	N	%
**Hair thickness**	19097																	
Thick	6104	31.96%	4030	66.02%	3598	58.94%	3199	52.41%	2622	42.96%	1357	22.23%	978	16.02%	899	14.73%	831	13.61%
Thin	5401	28.28%	3618	66.99%	3248	60.14%	2885	53.42%	2414	44.70%	1186	21.96%	866	16.03%	792	14.66%	688	12.74%
				0.273		0.1936		0.2798		0.06045		0.72525		0.9862		0.9228		0.166160
**Hair type**	9470																	
Dry	7212	76.16%	4636	64.28%	4173	57.86%	3720	51.58%	3066	42.51%	1676	23.24%	1253	17.37%	1124	15.59%	1038	14.39%
Oily	2258	23.84%	1547	68.51%	1352	59.88%	1222	54.12%	1041	46.10%	543	24.05%	389	17.23%	376	16.65%	330	14.61%
				0.0002		0.090		0.0351		0.00266		0.4284		0.8727		0.2256		0.79340
**Hair damage**	19096																	
Damaged	8266	43.29%	6156	74.47%	5091	61.59%	4781	57.84%	4133	50.00%	1957	23.68%	1502	18.17%	1344	16.26%	1195	14.46%
Not damaged	10830	56.71%	6711	61.97%	6329	58.44%	5676	52.41%	4606	42.51%	2123	19.60%	1483	13.69%	1373	12.68%	1273	11.75%
				<0.0001		<0.0001		<0.0001		<0.0001		<0.0001		<0.0001		<0.0001		<0.0001
Damaged male	1541	29.99%	1110	72.03%	917	59.51%	903	58.60%	859	55.74%	340	22.06%	276	17.91%	256	16.61%	265	17.20%
Damaged female	6723	46.96%	5046	75.06%	4174	62.09%	3878	57.68%	3274	48.70%	1616	24.04%	1225	18.22%	1087	16.17%	929	13.82%
				0.0153		0.0646		0.5300		0.0000		0.1073		0.8037		0.6980		0.0008
CURL degree																		
Straight or wavy	11236	58.84%	7527	66.99%	6486	57.73%	6074	54.06%	4906	43.66%	2196	19.54%	1655	14.73%	1450	12.90%	1323	11.77%
Curly	4340	22.73%	2912	67.10%	2865	66.01%	2499	57.58%	2201	50.71%	1088	25.07%	803	18.50%	732	16.87%	641	14.77%
Kinky	3521	18.44%	2182	61.97%	1889	53.65%	1700	48.28%	1468	41.69%	727	20.65%	467	13.26%	477	13.55%	457	12.98%
	19,097			<0.0001		<0.0001		<0.0001		<0.0001		<0.0001		<0.0001		<0.0001		<0.0001

**Note**: P-values that are significant are shown in red [P-value <0.05].

A 64.3% of individuals with dry hair had high stress levels in their personal life vs 68.5% of those with oily hair (p<10^−3^). Moreover “lack of sleep”, and “ stress level in professional life” were significantly higher in oily hair individuals (54.1% vs 51.6%, p=0.03, and 46.1% vs 42.5%, p=0.002). Hair damage state and different curl degrees also had significantly different impact on daily life (Table 3).

Our results show that the highest proportion of damaged hair was in China (44.8%) and Japan (44.6%) and the lowest in France (35.4%). Among those with damaged hair, the highest level of stress in personal life was in Japan (81.1%), stress in professional life in India (68.1%), and impact on sleep in India (66.5%) (Table 4). The highest proportion of dry hair was in Japan (44.8%) and USA (40.5%) and lowest in China (33%). Among those with dry hair, the highest level of stress in personal life was in Japan (84.4%), stress in professional life in India (63.9%), and impact on sleep in India (68.3%). The highest proportion of oily hair was in China (14.3%) and India (13.8%) and lowest in France (7.7%). Among those with oily hair, the highest level of stress in personal life was in Japan (84.4%), stress in professional life in India (63.9%), and impact on sleep in India (68.8%) (Table 4).

**Table 4:** Impact on Daily Life per Country in Those with Damaged, Dry, and Oily Hair

				Stress Level in Personal Life		Tiredness		Lack Sleep		Stress Level in Professional Life		Lack Energy		Lack Confidence		Feel Bad About Myself		Lack Cheerfulness	
Hair damage				N	%	N	%	N	%	N	%	N	%	N	%	N	%	N	%
	19,461	8266	42.5%	5431	65.7%	4787	57.91%	4352	52.65%	3640	44.04%	1760	21.29%	1308	15.82%	1170	14.15%	1088	13.16%
Brazil	2073	882	42.5%	572	64.9%	615	69.73%	450	51.02%	485	54.99%	272	30.84%	160	18.14%	196	22.22%	140	15.87%
**China**	2603	1166	44.8%	808	69.3%	490	42.02%	532	45.63%	356	30.53%	202	17.32%	137	11.75%	150	12.86%	121	10.38%
France	1617	573	35.4%	420	73.3%	401	69.98%	348	60.73%	135	23.56%	82	14.31%	76	13.26%	57	9.95%	22	3.84%
India	2960	1302	44.0%	829	63.7%	888	68.20%	866	66.51%	887	68.13%	265	20.35%	251	19.28%	196	15.05%	220	16.90%
Indonesia	2255	1000	44.3%	445	44.5%	533	53.30%	484	48.40%	323	32.30%	266	26.60%	183	18.30%	137	13.70%	166	16.60%
**Japan**	451	208	46.1%	170	81.7%	57	27.40%	77	37.02%	45	21.63%	43	20.67%	78	37.50%	37	17.79%	27	12.98%
Mexico	2292	1023	44.6%	746	72.9%	967	94.53%	550	53.76%	510	49.85%	147	14.37%	104	10.17%	96	9.38%	85	8.31%
South Africa	2698	1090	40.4%	711	65.2%	549	50.37%	468	42.94%	404	37.06%	236	21.65%	179	16.42%	169	15.50%	179	16.42%
USA	2512	1022	40.7%	730	71.4%	618	60.47%	577	56.46%	495	48.43%	247	24.17%	140	13.70%	132	12.92%	128	12.52%
				**Stress Level in Personal Life**		**Tiredness**		**Lack Sleep**		**Stress Level in Professional Life**		**Lack Energy**		**Lack Confidence**		**Feel Bad About Myself**		**Lack Cheerfulness**	
**Hair type**				**N**	**%**	**N**	**%**	**N**	**%**	**N**	**%**	**N**	**%**	**N**	**%**	**N**	**%**	**N**	**%**
**Dry**	19461	7212	37.1%	4774	66.2%	4175	57.89%	3836	53.19%	3175	44.02%	1518	21.05%	1119	15.52%	987	13.69%	918	12.73%
Brazil	2073	717	34.6%	454	63.3%	499	69.60%	354	49.37%	397	55.37%	228	31.80%	124	17.29%	143	19.94%	113	15.76%
**China**	2603	860	33.0%	577	67.1%	353	41.05%	389	45.23%	265	30.81%	156	18.14%	121	14.07%	114	13.26%	102	11.86%
France	1617	564	34.9%	428	75.9%	407	72.16%	343	60.82%	133	23.58%	72	12.77%	81	14.36%	62	10.99%	23	4.08%
India	2960	1033	34.9%	666	64.5%	712	68.93%	706	68.34%	707	68.44%	205	19.85%	184	17.81%	153	14.81%	166	16.07%
Indonesia	2255	833	36.9%	397	47.7%	428	51.38%	410	49.22%	262	31.45%	216	25.93%	155	18.61%	117	14.05%	133	15.97%
**Japan**	451	202	44.8%	169	83.7%	63	31.19%	74	36.63%	50	24.75%	48	23.76%	76	37.62%	41	20.30%	33	16.34%
Mexico	2292	903	39.4%	645	71.4%	859	95.13%	493	54.60%	464	51.38%	129	14.29%	85	9.41%	88	9.75%	73	8.08%
South Africa	2698	1083	40.1%	713	65.8%	541	49.95%	502	46.35%	405	37.40%	236	21.79%	166	15.33%	153	14.13%	162	14.96%
USA	2512	1017	40.5%	725	71.3%	618	60.77%	565	55.56%	492	48.38%	228	22.42%	127	12.49%	116	11.41%	113	11.11%
				**Stress Level in Personal Life**		**Tiredness**		**Lack Sleep**		**Stress Level in Professional Life**		**Lack Energy**		**Lack Confidence**		**Feel Bad About Myself**		**Lack Cheerfulness**	
**Hair type**				**N**	**%**	**N**	**%**	**N**	**%**	**N**	**%**	**N**	**%**	**N**	**%**	**N**	**%**	**N**	**%**
**Oily**	19461	2258	11.6%	1495	66.2%	1327	58.77%	1226	54.30%	1043	46.19%	459	20.33%	350	15.50%	320	14.17%	292	12.93%
Brazil	2073	227	11.0%	150	66.1%	162	71.37%	124	54.63%	132	58.15%	60	26.43%	36	15.86%	47	20.70%	34	14.98%
**China**	2603	373	14.3%	269	72.1%	169	45.31%	177	47.45%	120	32.17%	65	17.43%	43	11.53%	40	10.72%	37	9.92%
France	1617	125	7.7%	97	77.6%	92	73.60%	76	60.80%	30	24.00%	14	11.20%	14	11.20%	14	11.20%	3	2.40%
India	2960	407	13.8%	248	60.9%	288	70.76%	277	68.06%	260	63.88%	82	20.15%	83	20.39%	67	16.46%	74	18.18%
Indonesia	2255	283	12.5%	133	47.0%	164	57.95%	140	49.47%	115	40.64%	65	22.97%	49	17.31%	35	12.37%	41	14.49%
**Japan**	451	45	10.0%	38	84.4%	12	26.67%	18	40.00%	10	22.22%	12	26.67%	21	46.67%	11	24.44%	8	17.78%
Mexico	2292	264	11.5%	197	74.6%	254	96.21%	144	54.55%	141	53.41%	37	14.02%	28	10.61%	34	12.88%	21	7.95%
South Africa	2698	281	10.4%	186	66.2%	130	46.26%	126	44.84%	98	34.88%	61	21.71%	42	14.95%	40	14.23%	41	14.59%
USA	2512	253	10.1%	177	70.0%	144	56.92%	144	56.92%	137	54.15%	63	24.90%	34	13.44%	32	12.65%	33	13.04%

**Note**: In red, the cumulative values for the 9 countries involved in the project.

The highest prevalence of straight or wavy hair was in China (86.6%) and Japan (84.3%). Among those with straight or wavy hair, the highest stress levels in personal life was in Japan (81.3%) and the lowest in Indonesia (45.5%) and Brazil (58.6%). The highest impact on sleep was in India (68.2%) and France (60%) and the lowest in Japan (37.4%). The highest stress level in professional life was in India (68.4%) and the lowest in Japan (23.4%) and France (22.6%). The highest prevalence of curly hair was in Brazil (42.6%) and lowest in China (5.5%). Among those with curly hair, the highest stress level in personal life was in France, stress in professional life in India (64.2%), and impact on sleep in France (68.5%). The highest prevalence of kinky hair was in South Africa (59.4%) and lowest in Japan (0.9%) (Table 5).

**Table 5:** Impact on Daily Life per Country According to the Degree of Curl

				**1**		**5**		**5**		**5**		**1**		1		1		1	
				**Stress Level in Personal Life**		**Tiredness**		**Lack Sleep**		**Stress Level in Professional Life**		**Lack Energy**		**Lack Confidence**		**Feel Bad About Myself**		**Lack Cheerfulness**	
**CURL degree**				**N**	**%**	N	%	**N**	**%**	**N**	**%**	**N**	**%**	**N**	**%**	**N**	**%**	**N**	**%**
Straight or wavy	19461	11,236	57.7%	7527	67.0%	6486	57.73%	6074	54.06%	4906	43.66%	2196	19.54%	1655	14.73%	1450	12.90%	1323	11.77%
Brazil	2073	476	23.0%	279	58.6%	323	67.86%	234	49.16%	278	58.40%	135	28.36%	82	17.23%	100	21.01%	74	15.55%
China	2603	2255	86.6%	1580	70.1%	960	42.57%	1021	45.28%	716	31.75%	390	17.29%	261	11.57%	279	12.37%	229	10.16%
France	1617	1142	70.6%	859	75.2%	817	71.54%	685	59.98%	258	22.59%	151	13.22%	156	13.66%	126	11.03%	38	3.33%
India	2960	2108	71.2%	1341	63.6%	1429	67.79%	1437	68.17%	1442	68.41%	358	16.98%	307	14.56%	246	11.67%	299	14.18%
Indonesia	2255	1620	71.8%	737	45.5%	822	50.74%	772	47.65%	523	32.28%	391	24.14%	256	15.80%	195	12.04%	235	14.51%
Japan	451	380	84.3%	309	81.3%	121	31.84%	142	37.37%	89	23.42%	98	25.79%	148	38.95%	75	19.74%	64	16.84%
Mexico	2292	1435	62.6%	1060	73.9%	1426	99.37%	789	54.98%	756	52.68%	199	13.87%	147	10.24%	141	9.83%	108	7.53%
South Africa	2698	749	27.8%	559	74.6%	436	58.21%	400	53.40%	323	43.12%	236	31.51%	166	22.16%	161	21.50%	163	21.76%
USA	2512	1071	42.6%	803	75.0%	657	61.34%	594	55.46%	521	48.65%	238	22.22%	132	12.32%	127	11.86%	113	10.55%
	19,461	11,236		7527	617.8%	6991	55127	6074	47146	4906	38131	2196	19339	1655	15651	1450	13104	1323	1144
				**Stress Level in Personal Life**		**Tiredness**		**Lack Sleep**		**Stress Level in Professional Life**		**Lack Energy**		**Lack Confidence**		**Feel Bad About Myself**		**Lack Cheerfulness**	
**CURL degree**				**N**	**%**	N	%	**N**	**%**	**N**	**%**	**N**	**%**	**N**	**%**	**N**	**%**	**N**	**%**
Curly	19461	4340	22.3%	2912	67.1%	2865	66.01%	2499	57.58%	2201	50.71%	1088	25.07%	803	18.50%	732	16.87%	641	14.77%
Brazil	2073	884	42.6%	609	68.9%	645	72.96%	494	55.88%	524	59.28%	308	34.84%	163	18.44%	187	21.15%	152	17.19%
China	2603	142	5.5%	101	71.1%	77	54.23%	70	49.30%	51	35.92%	28	19.72%	25	17.61%	24	16.90%	25	17.61%
France	1617	346	21.4%	285	82.4%	278	80.35%	237	68.50%	118	34.10%	47	13.58%	56	16.18%	47	13.58%	15	4.34%
India	2960	806	27.2%	472	58.6%	591	73.33%	548	67.99%	518	64.27%	213	26.43%	225	27.92%	183	22.70%	183	22.70%
Indonesia	2255	583	25.9%	281	48.2%	330	56.60%	292	50.09%	203	34.82%	167	28.64%	131	22.47%	92	15.78%	99	16.98%
Japan	451	45	10.0%	37	82.2%	11	24.44%	16	35.56%	8	17.78%	9	20.00%	17	37.78%	9	20.00%	6	13.33%
Mexico	2292	596	26.0%	448	75.2%	564	94.63%	327	54.87%	309	51.85%	84	14.09%	61	10.23%	67	11.24%	47	7.89%
South Africa	2698	283	10.5%	200	70.7%	154	54.42%	147	51.94%	124	43.82%	86	30.39%	57	20.14%	58	20.49%	53	18.73%
USA	2512	655	26.1%	479	73.1%	414	63.21%	368	56.18%	346	52.82%	146	22.29%	68	10.38%	65	9.92%	61	9.31%
				**Stress Level in Personal Life**		**Tiredness**		**Lack Sleep**		**Stress Level in Professional Life**		**Lack Energy**		**Lack Confidence**		**Feel Bad About Myself**		**Lack Cheerfulness**	
**CURL degree**				**N**	**%**	N	%	**N**	**%**	**N**	**%**	**N**	**%**	**N**	**%**	**N**	**%**	**N**	**%**
Kinly	19461	3521	18.1%	2181	61.9%	1888	53.62%	1700	48.28%	1467	41.66%	727	20.65%	467	13.26%	477	13.55%	457	12.98%
Brazil	2073	707	34.1%	426	60.3%	491	69.45%	378	53.47%	395	55.87%	229	32.39%	132	18.67%	152	21.50%	122	17.26%
China	2603	135	5.2%	80	59.3%	44	32.59%	61	45.19%	42	31.11%	24	17.78%	18	13.33%	18	13.33%	16	11.85%
France	1617	120	7.4%	86	71.7%	80	66.67%	81	67.50%	34	28.33%	16	13.33%	12	10.00%	12	10.00%	8	6.67%
India	2960	28	0.9%	12	42.9%	20	71.43%	21	75.00%	23	82.14%	8	28.57%	7	25.00%	6	21.43%	5	17.86%
Indonesia	2255	31	1.4%	15	48.4%	23	74.19%	22	70.97%	12	38.71%	13	41.94%	7	22.58%	8	25.81%	9	29.03%
Japan	451	4	0.9%	4	100.0%	2	50.00%	2	50.00%	0	0.00%	1	25.00%	1	25.00%	0	0.00%	0	0.00%
Mexico	2292	221	9.6%	152	68.8%	168	76.02%	101	45.70%	99	44.80%	31	14.03%	26	11.76%	26	11.76%	19	8.60%
South Africa	2698	1602	59.4%	982	61.3%	734	45.82%	652	40.70%	532	33.21%	240	14.98%	179	11.17%	172	10.74%	181	11.30%
USA	2512	673	26.8%	425	63.2%	381	56.61%	382	56.76%	331	49.18%	165	24.52%	85	12.63%	83	12.33%	97	14.41%

**Notes**: In red, the cumulative values for the 9 countries involved in the project.

## Discussion

This is the first multinational study that assessed hair type and characteristics and impact on daily life. Even subtle manipulations of a person’s hair can alter people’s perception of his age, attractiveness, and health.^9^ Most dermatological literature on hair has focused on effect of hair loss on people’s daily lives, yet none has assessed the impact of hair types on quality of life. Hair that is healthy, strong, and shiny signals overall physical health. Conversely, hair that is dry, damaged, and thin is perceived to be unhealthy and related to an illness, which makes the person less attractive.^9^ Since people tend to be seduced by healthy, young, and attractive individuals, well-groomed and good-looking hair may signal these parameters.^9^,^10^ However, our study showed that around a quarter of males and a third of females reported thin hair, and a significant difference as noted in those aged ≥40 y.o compared to those <40 y.o. Moreover, around a third of males and 40% of females reported dry hair, with a significantly higher proportion in those ages 40 and above. With regard to damaged hair, it is reported in around half of females and a third of males, with surprisingly, a higher proportion in those below the age of 40 compared to those aged 40 and above. A significantly higher proportion of females reported their hair being “frizzy due to humidity”, “dull without shine”, “with split ends”, or “difficult to smoothen”. Difference between males and females was noted regarding hair having “badly defined curls”, “too much volume”, and “low density”. The proportion of curly hair in females was significantly higher than in males. Kinky hair was significantly more frequent in males and in those <40 y.o.

Thick hair was more frequent in African followed by Hispanic, European, and finally Asian ethnicity. On the contrary, thin hair was more frequent in European (over a third of individuals) followed by Hispanic, African, and lastly Asian ethnicity. Dry hair was significantly higher in African ethnicity followed by Hispanic, Asian, and lastly European ethnicity. Asian hair is known for exhibiting the strongest mechanical properties and is known for its straightness, and large diameter which is attributed to the cuticle layer being thicker than that in Caucasians with more compact cuticle cells.^11^,^12^ Surprisingly, the highest proportion of damaged hair was noted in Asian followed by Hispanic, European, and lastly African ethnicity.

Straight hair was most frequent in Asian ethnicity, wavy hair in European and Asian ethnicity, curly hair in Hispanic and Asian ethnicity, while kinky hair was most frequent in Hispanic ethnicity.

Impact on daily life in patients with alopecia areata and androgenetic alopecia has been studied quite extensively^13–20^ and reflects the impairment that hair problems can have, which is a greater impairment than that of other common dermatoses like acne vulgaris. Androgenetic alopecia is also associated with impairment in emotions, which is greater than in the function and symptom dimensions.^13^ However, comparably, very little is known on the effect of hair types on an individual’s personal well-being. Our study showed that individuals with damaged hair tend to be significantly more tired, have higher stress levels on personal and professional lives and lack of sleep. Moreover, they tend to be less energetic, confident, cheerful, feel less good about themselves. No significant difference was noted between those who have thick vs thin hair regarding stress levels, lack of sleep, tiredness, work–life balance, confidence, cheerfulness, and feeling good about themselves. However, those with oily hair tend to be more stressed and those with dry hair tend to have less work–life balance. Damaged hair in males and females had a different impact on daily life. Males with damaged hair were more likely to have stress in their professional life and to lack cheerfulness. On the contrary, females with damaged hair were more likely to have stress in their personal life.

Hair types and characteristics on daily life differed significantly among countries. Moreover, their impact on daily life also differed among countries. This might highly be attributed to differences in perception of beauty among cultures.

Our results show that the highest proportion of damaged hair was in China and the lowest in France. The highest proportion of dry hair was in Japan and lowest in China. The highest proportion of oily hair was in China and lowest in France. The highest prevalence of straight or wavy hair was in China. The highest prevalence of curly hair was in Brazil and lowest in China. The impact of curl degree on daily life differed between countries. Among those with straight or wavy hair, the highest stress level in personal life was in Japan and the lowest in Indonesia and Brazil. In fact, in Brazil, curly hair is celebrated and considered a symbol of beauty. This is probably due to the country’s culture and diverse population. It would be interesting to conduct future studies aiming to assess the exact relation between societal pressure and impact of curl degree and damage status on daily life. Despite the presence of a growing appreciation for curly hair in recent years in many countries, curly hair had significant impact on daily lives of individuals. Among those with curly hair, the highest stress level in personal life was in France, stress in professional life in India, and impact on sleep in France. Having more manageable hair that remains straight and capable of defying humidity has been a long-time desire among women across different ethnic groups. This is evident in the multitude of straightening techniques used across the globe.

## Conclusion

Our research shows that, while hair problems vary by age, gender, and ethnicity, they remain widespread. Given that hair is a prominent feature of physical appearance, our findings highlight the significant impact that hair type has on an individual’s daily life. Hair plays a crucial role in shaping self-image and identity, and understanding how hair characteristics affect both men and women’s daily experiences is essential. Our study demonstrates how individuals perceive themselves and how they are perceived by others, emphasizing the importance of hair in personal and social contexts. This insight can inform interventions aimed at enhancing body image and self-esteem. Additionally, our research contributes to a deeper understanding of the comprehensive impact of hair on people’s lives, which can lead to improvements in healthcare, social attitudes, and product development.

## Data Availability

The data that support the findings of this study are available from the corresponding author upon reasonable request.
